# Resolving the Taxonomic Status of* Chamelea gallina* and* C. striatula* (Veneridae, Bivalvia): A Combined Molecular Cytogenetic and Phylogenetic Approach

**DOI:** 10.1155/2017/7638790

**Published:** 2017-05-07

**Authors:** Daniel García-Souto, Vesa Qarkaxhija, Juan J. Pasantes

**Affiliations:** Departamento Bioquímica, Xenética e Inmunoloxía, Universidade de Vigo, Vigo, Spain

## Abstract

The striped venus clams* Chamelea gallina* and* C. striatula* are commercially important bivalves inhabiting European and North African coastal waters. The taxonomic status of these taxa has been the subject of debate for decades. In order to elucidate this issue, we generated 5S and 28S ribosomal RNA and H3 histone gene probes and mapped them by fluorescent in situ hybridization to the chromosomes of morphologically identified striped venus clams, collected from four geographically distant Atlantic and Mediterranean populations. The nucleotide variation at the three DNA markers, that is, the nuclear internal transcribed spacer 2 (ITS2), the mitochondrial cytochrome c oxidase subunit I (COI), and the large ribosomal subunit rRNA (16S) fragments, was also studied and the resultant phylogenetic trees were evaluated. Striking differences in both the chromosome distribution of these genes and the clustering of the samples on the phylogenetic trees observed provide clear evidence that* C. gallina* and* C. striatula* are separated species.

## 1. Introduction

The striped venus clams* Chamelea gallina* (Linnaeus, 1758) and* C. striatula* (da Costa, 1778) (Bivalvia: Veneridae), the only extant species in the genus* Chamelea* Mörch, 1853, are filter feeding, sand burrowing bivalves inhabiting European and North African coastal waters. Even though these taxa are commercially exploited and economically valuable, for decades, their taxonomic status has been a matter of debate [1] that is still not completely settled. The confused status of these taxa is partially due to the use of variable shell and siphon characteristics to identify them.* C. striatula* is distinguished from* C. gallina* by possessing a more pointed and ridged shell and longer siphons [2] and by residing in the Atlantic [3]. On the basis of shell and siphon morphologies,* C. striatula* and* C. gallina* have been considered members of a single polymorphic species, two geographically isolated subspecies, or two different species. The lack of modern genetic studies applied to differentiate* C. gallina* and* C. striatula* also contributes to their uncertain status. The genetic distances estimated after studying seven polymorphic enzymes in samples obtained from striped venus clam mixed beds in the south of Portugal [2], supporting their status as separated species, are, as yet, the only genetic study applied to these taxa.

Although shell shape analyses [4] and geometric morphometric methods [3] have helped in accurately predicting the origin of some* C. gallina* samples and differentiating* C. gallina* from* C. striatula*, establishing taxonomic boundaries within bivalves has always been hindered by a lack of diagnostic morphological features [5]. This is due to an extensive parallelism of interspecific variability as a result of convergent evolution in response to the same environment alongside a degree of phenotypic divergence among populations of a single taxon developed on different substrates. Therefore, it is inaccurate to rely on morphology alone to delimit species boundaries and further criteria have to be analyzed [5].

In comparison with many other groups of organisms, cytogenetic analyses in clams of the family Veneridae are scarce. All species studied to date show a diploid chromosome number of 2*n* = 38 [6, 7] and their karyotypes are mostly composed of chromosome pairs showing small differences in size and morphology, therefore making their accurate identification almost impossible [7]. As a resolution to this problem, along the last two decades, highly conserved repetitive DNA sequences have been used as probes in fluorescent in situ hybridization (FISH) experiments to identify chromosome pairs in bivalve species [8, 9], some of which were venus clams [7, 10–14]. Most commonly used DNA sequences for these types of studies are ribosomal RNA (rRNA) and histone genes as they are usually organized in tandems and clustered at one or more chromosomal positions. The employment of this approach in mussels of the* Mytilus edulis* species complex has allowed for differentiating* M. edulis* and* M. galloprovincialis* from* M. trossulus* by the chromosomal location of one of the H3 histone gene clusters and the number and location of the major rRNA gene (rDNA) clusters [15].

Chromosomal studies on striped venus clams are rarer than in other venerid clams. To the best of our knowledge, there is no published karyological information for* C. striatula*. For* C. gallina*, information is mostly limited to two reports describing chromosome number and karyotype composition and the location of major rDNA, 5S rDNA, and H3 histone gene clusters [7, 16].

In regard to molecular phylogenetic analysis, many studies have utilized the mitochondrial cytochrome c oxidase subunit I (COI) gene as the standard most accurate marker for delimitating and clarifying the taxonomic status of animal species, including venerid clams [17]. The mitochondrial 16S rRNA gen has also been successfully employed for species identification in clams [17, 18]. These mitochondrial sequences are known to evolve faster than nuclear genes, making them ideal tools for detecting differences among closely related species and among populations within a species. In addition, the sequences of the internal transcribed spacer 2 (ITS2) of the 18S-5.8S-28S nuclear rDNA, and their corresponding secondary structures of the ITS2 rRNAs, have also proved suitable for assessing phylogenetic and phylogeographic relationships among many animal taxa, some of them venerid clams [19].

In order to contribute to a better understanding of the evolutionary history and diversification within the genus* Chamelea*, we used an integrated approach based, on the one hand, on the comparison of the karyotypes constructed after mapping by FISH three tandemly repeated gene families (5S rDNA, 28S rDNA, and H3 histone genes) and, on the other hand, on the molecular phylogenies obtained from one nuclear (ITS2) and two mitochondrial (COI and 16S rRNA genes) sequences in striped venus clams collected from two Atlantic populations (Ría de Pontevedra, NW Spain; Gulf of Cádiz, S Spain) and two Mediterranean ones (Gulf of Valencia, E Spain; Adriatic Sea, E Italy). The genetic evidence obtained in this work confirms the consideration of* C. gallina* and* C. striatula* as separated species.

## 2. Materials and Methods

### 2.1. Sampling and Identification

**Figure 1:**
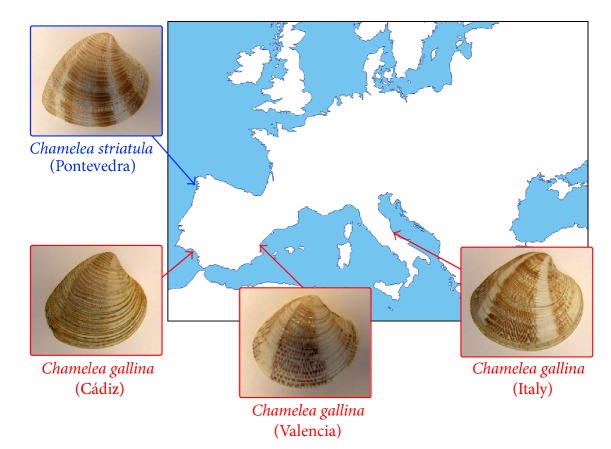
Collection localities and representative shells of the striped venus analyzed.

Specimens of striped venus clams were collected (Figure 1) from different localities in Ría de Pontevedra (Atlantic coast, NW Spain) and identified as* C. striatula*, through their morphological attributes. Other striped venus clam samples, identified as* C. gallina*, were collected from natural beds in Valencia (Gulf of Valencia, Mediterranean coast, E Spain) and from local market places in coastal towns of the Gulf of Cádiz (Atlantic coast, S Spain) and the Adriatic Sea (Mediterranean coast, E Italy). The nomenclature utilized for these taxa follows the World Register of Marine Species (WoRMS) database (http://www.marinespecies.org/). In all cases, striped venus clams were transported alive to the lab, maintained in tanks of 5 L filtered seawater at 18 ± 1°C, and fed on microalgae to promote somatic growth.

### 2.2. Chromosome Preparation

Chromosome spreads were obtained as previously published [20, 21]. Following an in vivo colchicine (0.005%, 10 h) treatment, striped venus clams were dissected and gills and gonads were immersed in diluted sea water (50%, 20 min; 25%, 20 min). After fixation in ethanol/acetic acid (3 : 1; 3 times 20 min), pieces of tissue were disaggregated in 60% acetic acid and the cell suspensions spread onto warm microscope slides [22, 23].

### 2.3. DNA Extraction and PCR Amplification

DNA was extracted from adductor muscles using a phenol-chloroform-isoamyl alcohol method [24]. A fragment of the mitochondrial COI gene was amplified by PCR employing the standard barcoding primers* LCO1490* and* HCO2198* [25]. A fragment of the mitochondrial 16S rRNA gene was amplified by means of primers* 16L29* [26] and* 16SBr* [27]. The complete ITS2 of the major rDNA was amplified using primers* ITS3* and* ITS4 *[28]. On FISH mapping purposes, universal primers* LR10R* and* LR12* retrieved from Vilgalys lab website [29] were used to amplify a fragment of the 28S rDNA. Amplifications of the entire 5S rDNA repeat and the H3 histone genes used primers described by Pérez-García et al. [22, 23] and Giribet and Distel [30], respectively.

DNA sequences were amplified in a GeneAmp PCR system 9700 (Applied Biosystems) in 50 *μ*L solutions containing 125 ng of genomic DNA, 50 *μ*M each dNTP, 50 *μ*M each primer, 1xPCR buffer, 15 *μ*M MgCl_2_, and 5 U of JumpStart™ Taq DNA Polymerase (Sigma). Amplifications included an initial denaturation step at 95°C (2 min), 35 amplification cycles (Supplementary Table  1, in Supplementary Material available online at https://doi.org/10.1155/2017/7638790), and a final extension at 72°C (5 min). PCR products were examined by electrophoresis on 2% agarose gels.

### 2.4. DNA Sequencing and Phylogenetic Analysis

Amplified mitochondrial 16S rRNA and COI genes and nuclear ITS2 rDNA sequences were purified (FavorPrep™ GEL/PCR Purification Kit, Favorgen) and sequenced (CACTI, University of Vigo) in both directions in an ABI PRISM 3730 Genetic Analyzer (Applied Biosystems) using a BigDye Terminator v. 3.1 Cycle Sequencing Kit (Applied Biosystems). The sequences were edited with BioEdit v. 7.1.11 [31] and aligned with Muscle set to default parameters using MEGA7 [32]. Sequence similarity searches were performed using the Basic Local Alignment Search Tool (BLAST) algorithm, available at the National Center for Biotechnology Information (NCBI) (http://www.ncbi.nlm.nih.gov/blast). The MegaBLAST algorithm set to default parameters was employed against both NCBI nucleotide collection and NCBI nucleotide collection and Barcode of Life Data System (BOLD) databases. After removing primers, maximum-likelihood (ML) phylogenetic analyses were performed. The best-fit nucleotide substitution models were selected (COI gene: JC + G; 16S rRNA gene: HKY+G; ITS2: T92) by the AIC criterion employing JModelTest 2 [33, 34]. ML reliability was assessed with 500 bootstrap replicates. All phylogenetic analyses were performed on MEGA7 [32]. Nucleotide diversity (pi) and uncorrected pairwise* p*-distances were estimated using DnaSP v. 5 [35] and MEGA7 [32], respectively.

### 2.5. Fluorescence In Situ Hybridization (FISH)

Single, double, and sequential FISH experiments using 5S and 28S rDNA and H3 histone gene probes were performed on metaphase chromosome spreads obtained from striped venus clams collected in all four regions. Biotin and digoxigenin labeled probes were generated either directly by PCR or by nick translation [36]. Chromosome slides were digested with RNase and pepsin before denaturation (70°C, 2 min) and hybridized overnight at 37°C. Biotin was detected with fluorescein isothiocyanate (FITC) conjugated avidin and biotinylated anti-avidin (Vector) whereas digoxigenin was detected with anti-digoxigenin antibodies conjugated with tetramethylrhodamine isothiocyanate (TRITC) (Sigma). Chromosome slides were counterstained with 4′-6-diamidino-2-phenylindole (DAPI, 0.14 *μ*g/mL in 2xSSC) and mounted with antifade (Vectashield, Vector).

Chromosome preparations were examined with a Nikon Eclipse-800 microscope equipped with an epifluorescence system [36]. Separated images for each fluorochrome were recorded and pseudocolored using a DS-Qi1Mc CCD camera (Nikon) controlled by the NIS-Elements software (Nikon). Merging of the images was performed with Adobe Photoshop.

## 3. Results

### 3.1. Karyotypes and Chromosomal Mapping of rDNA and H3 Histone Gene Clusters

**Figure 2:**
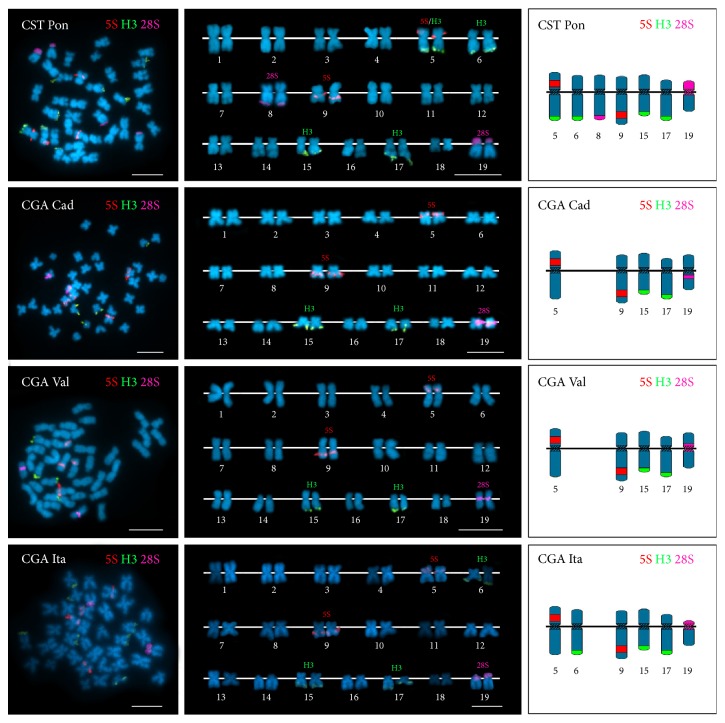
FISH mapping of 5S rRNA (5S, red), 28S rRNA (28S, magenta), and H3 histone gene (H3, green) probes to mitotic metaphase chromosomes of striped venus clams* Chamelea striatula* from Pontevedra (CST Pon) and* Chamelea gallina* from Cádiz (CGA Cad), Valencia (CGA Val), and Italy (CGA Ita) counterstained with DAPI. The corresponding karyotypes and schematic representations of the signal bearing chromosomes are also included. Note that all chromosome pairs present a single signal with the exception of CST Pon chromosome pair 5 that bears both 5S rDNA and H3 histone gene clusters. Scale bars, 5 *μ*m.

All striped venus clams analyzed showed mitotic metaphase plates presenting 38 chromosomes (Figure 2). The karyotypes constructed for the four striped venus clam populations (Figure 2) were roughly similar for 18 of the chromosome pairs (7 metacentric, 6 meta/submetacentric, 2 submetacentric, and 3 subtelocentric) but the remaining one (number 19 for comparative purposes) showed morphological differences. This chromosome pair was subtelocentric in both* C. striatula* and the Italian* C. gallina* and metacentric in the two Spanish* C. gallina* populations.

FISH mapping of 5S rDNA probes showed intercalary signals located in two metacentric chromosome pairs (5p and 9q) in all striped venus clams regardless of origin and whether they were morphologically identified as* C. gallina* or* C. striatula* (Figure 2). In contrast, the number and the distribution of the signals corresponding to both 28S rDNA and H3 histone gene probes evinced some differences. All striped venus clam specimens presented at minimum a single 28S rDNA signal situated in the neighborhood of the centromere on chromosome pair 19; whereas this is the only signal in* C. gallina*, an additional signal subterminal to 8q appeared in* C. striatula* (Figure 2). Likewise, although H3 histone gene probes were subterminal to 15q and 17q in all striped venus clams, further signals were present in both the Italian* C. gallina* population (6q) and in* C. striatula *(6q and 5q).

Double-color FISH mapping using 5S rDNA and H3 histone gene probes also confirmed the presence of signals for both probes on* C. striatula* chromosome pair 5. This chromosome pair only bears 5S rDNA clusters in* C. gallina*.

In all four populations, variation in signal patterning was low, and aberrance was reduced to a pericentric inversion or an additional 28S rDNA signal in four of the 55* C. striatula* specimens and one additional 28S rDNA signal in two of the 28 specimens of the Italian* C. gallina*.

### 3.2. DNA Sequence Variation and Genetic Divergence

**Figure 3:**
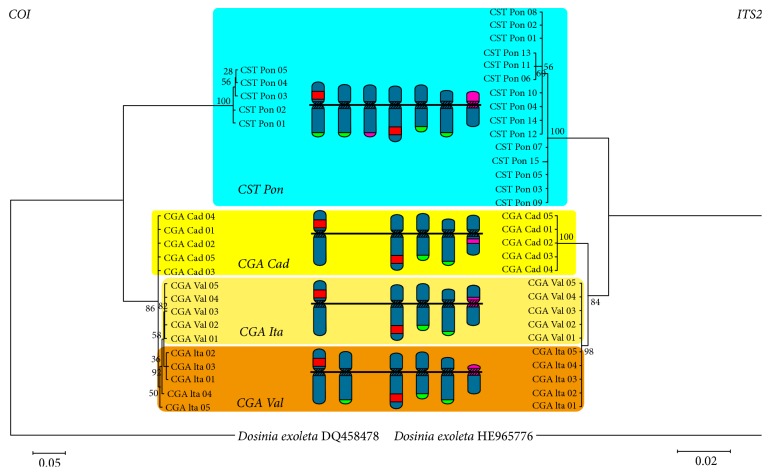
Maximum-likelihood trees based on mitochondrial COI gene and nuclear ITS2 sequences of striped venus clams using* Dosinia exoleta* as outgroup together with a schematic representation of the main chromosomal differences. Numbers in internal nodes indicate maximum-likelihood bootstrap support values (500 replicates).

A fragment of 629 bp, excluding primers, of the mitochondrial COI gene was sequenced for 20 striped venus clams, five per population (GenBank acc. numbers KY547747 to KY547766). The sequences showed 89 polymorphic sites and 78 differentially fixed mutations. The nucleotide diversity was 0.0556 (Table 1) and the genetic distance between taxa was 13.20% (Table 2). The comparison of these sequences with those stored in GenBank showed that our* C. striatula* sequences coincided in a 99% with those from North Sea* C. gallina* samples [37]. The* C. gallina* COI sequences displayed a high level of homology (98%) with samples from the Adriatic Sea (GenBank acc. numbers DQ458474 and KR078004) and a market place in the Canary Islands (GenBank acc. number DQ184835). Pairwise genetic distances between populations inferred from the mitochondrial COI gene sequences (Table 2) showed that* C. striatula* is clearly distinct from the three* C. gallina* populations. Likewise, the ML tree recovered by MEGA on these sequences (using* Dosinia exoleta* as outgroup) revealed two well supported clades, one formed by the specimens morphologically identified as* C. striatula* and the other by the specimens identified as* C. gallina* with bootstrap values of 100% and 86%, respectively (Figure 3).

The sequenced 16S rRNA gene fragments were 468 bp long in the specimens collected from Pontevedra* (C. striatula)* and 466 bp long in all* C. gallina* specimens (GenBank acc. numbers KY547767 to KY547786). The difference in length was due to six between-taxa gaps: two single nucleotide and one dinucleotide insertions in* C. striatula* and two single nucleotide insertions in* C. gallina*. There were 51 polymorphic sites, 48 of them taxa specific, providing a genetic distance between taxa of 11.44%. The nucleotide diversity was 0.0443 (Table 1). These 16S rRNA gene sequences were also compared with the six sequences stored in GenBank. The* C. striatula* sequences were identical to sequences from England (GenBank acc. numbers DQ280041 and KX713203) whereas those from* C. gallina* matched the sequences of specimens from Turkey (GenBank acc. number AM085110), Adriatic Sea (GenBank acc. number AJ548762), NW Spain (GenBank acc. number JF808193), and a market in the Canary Islands (GenBank acc. number DQ184735). Again, both the pairwise distances (Table 2) and the ML tree recovered (not shown) differentiated specimens belonging to the two taxa (bootstrap values of 100% and 72%).

The amplified ITS2 fragments (GenBank acc. numbers KY508254 to KY508283) were the most inconsistent in length. While all specimens of the Mediterranean populations of* C. gallina* displayed 498 bp long fragments, in the Atlantic specimens, the ITS2 was 496 bp long; these differences in length were a result of a dinucleotide insertion (or deletion) and six nucleotide substitutions. In contrast, five specimens of the* C. striatula* population displayed 498 bp long ITS2 fragments whereas the remaining 10 exhibited 495 bp long fragments, due to a trinucleotide insertion and two point mutations. Sequence analysis of the ITS2 for all striped venus clams revealed 34 polymorphic sites and, excluding gaps, 13 differentially fixed mutations between the two taxa. The nucleotide diversity was 0.0202 (Table 1) and the genetic distance between taxa was 3.52% (Table 2). While the Atlantic* C. gallina* sequences obtained in this work were identical to those from Tyrrhenian Sea specimens (GenBank acc. numbers HE965773 and HE965774), the two Mediterranean populations ITS2 sequences coincided with those from Adriatic Sea samples (GenBank acc. numbers HE965771 and HE965772). No* C. striatula* ITS2 sequences were found in GenBank. The pairwise genetic distances between populations inferred from nuclear ITS2 sequences (Table 2) also depicted that* C. striatula* is clearly separated from the three* C. gallina* populations and the ML tree also recovered two clades with bootstrap values of 100% and 84% (Figure 3).

In addition, the predicted folding shapes of the* C. gallina* ITS2 rRNAs (Supplementary Figure 1) were identical to those previously published for the same species [19]; those for* C. striatula* were concordant with the structure proposed for Veneridae [19]. Most point mutations detected (17 of a total of 20), including those differentiating taxa and populations, were clustered on the DIV-DVI stems, the less conserved area in terms of primary sequence [19].

## 4. Discussion and Conclusion

The taxonomic status of the striped venus clams* C. gallina* and* C. striatula* has been a matter of debate for decades [1]. Although the World Register of Marine Species have recognized them as separated species since 2004 [38, 39], the distribution ranges of these taxa displayed in WoRMs, from the North Atlantic to the Eastern Mediterranean, still overlap in almost their entirety. Moreover, even as recently as last year, COI gene sequences obtained from North Sea samples, therefore* C. striatula*, were stored in GenBank under the specific name* C. gallina* [37]. In order to resolve this issue, we applied a molecular cytogenetic approach, from the perspective of chromosomal distribution of three gene families [7, 12–14], alongside comparing the sequences of mitochondrial and nuclear DNA markers, that are increasingly being utilized in phylogenetic studies [5, 17–19, 40, 41], to further insight and strengthen the evidence.

The diploid chromosome number of 2*n* = 38 obtained in this work is in accordance with those previously described for* C. gallina* [7, 16] and all other Veneridae studied to date [6–8, 12–14]. The karyotype compositions obtained were also fundamentally coincidental with that proposed for* C. gallina* [16].

Discordant with their conserved chromosome numbers, the species of Veneridae present clear differences in the number and distribution of rDNA and histone gene clusters on their chromosomes [7, 12–14]. These differences are also present in closely related, congeneric species and are usually accompanied with an almost complete absence of intraspecific variability [7, 12–14].

Our results demonstrated that this is also the case for the two striped venus clam taxa studied. The consistency in the 5S rDNA signal pattern and the mapping differences for both 28S rDNA and H3 histone gene signals found between* C. gallina* and* C. striatula* are of a similar magnitude to those reported for* Venus casina*/*V. verrucosa* and* Dosinia exoleta*/*D. lupinus* [7]. Conversely, although we found some mapping differences among* C. gallina* populations and among* C. striatula* specimens, these differences were comparatively narrower than those between taxa and similar to those found in other bivalve species [23, 36], thusly constituting the standard intraspecific variation.

The sequence data obtained also indicated that these two taxa are separated species. All individual ML trees recovered by MEGA on the COI gene, 16S rRNA gene, and ITS2 sequences (using* Dosinia exoleta* as outgroup) revealed two clearly separated, well supported clades, one formed by the specimens morphologically identified as* C. striatula* and the other by the specimens identified as* C. gallina*. Furthermore, the genetic distances between the* C. striatula* population and any of the* C. gallina* populations were indubitably higher than those between any two* C. gallina* populations for both mitochondrial and nuclear sequences. The magnitudes of these genetic distances were fully concordant with those previously reported for congeneric species of Veneridae, both for the mitochondrial COI gene [5] and the nuclear ITS2 [19] sequences analyzed.

In conclusion, the results obtained in this study after employing two mitochondrial and one nuclear DNA markers together with three chromosomal markers in four geographically distant populations of striped venus clams clearly demonstrate that* C. gallina* and* C. striatula* are well differentiated species.

## Supplementary Material

PCR conditions used in the amplification of COI, 16S rRNA gene, ITS2, histone H3 and 5S and 28S rRNA genes.

## Figures and Tables

**Table 1:** Nucleotide diversity (pi) of COI gene, 16S rDNA, and ITS2 sequences in *Chamelea gallina* and *Chamelea striatula.*

	COI gene		16S rDNA		ITS2	
	*n*	pi	*n*	pi	*n*	pi
*Chamelea striatula*						
CST Pon	5	0.0022	5	0.0000	15	0.0021
*Chamelea gallina*						
CST Cad	5	0.0000	5	0.0000	5	0.0000
CST Val	5	0.0000	5	0.0000	5	0.0000
CST Ita	5	0.0057	5	0.0035	5	0.0000
All CST	15	0.0060	15	0.0030	15	0.0063
All *Chamelea*	20	0.0556	20	0.0443	30	0.0202

**Table 2:** Pairwise *p*-distances between striped venus clam populations using mitochondrial COI and 16S rRNA genes and nuclear ITS2 sequences. Interspecific distances in bold.

Populations		COI gene	16S rDNA	ITS2
CST Pon	CGA Cad	**0.1304**	**0.1121**	**0.0396**
CST Pon	CGA Val	**0.1320**	**0.1164**	**0.0330**
CST Pon	CGA Ita	**0.1335**	**0.1147**	**0.0330**
CGA Cad	CGA Val	0.0095	0.0043	0.0132
CGA Cad	CGA Ita	0.0086	0.0047	0.0132
CGA Val	CGA Ita	0.0048	0.0022	0.0000
CST	CGA	**0.1320**	**0.1144**	**0.0352**
