# Supplementary figures and images for: Resolving the Taxonomic Status of Chamelea gallina and C. striatula (Veneridae, Bivalvia): A Combined Molecular Cytogenetic and Phylogenetic Approach
Source: Biomed Res Int. 2017 May 7;2017:7638790. doi: 10.1155/2017/7638790 (PMC5438835; doi:10.1155/2017/7638790)

CST Pon 495 bp

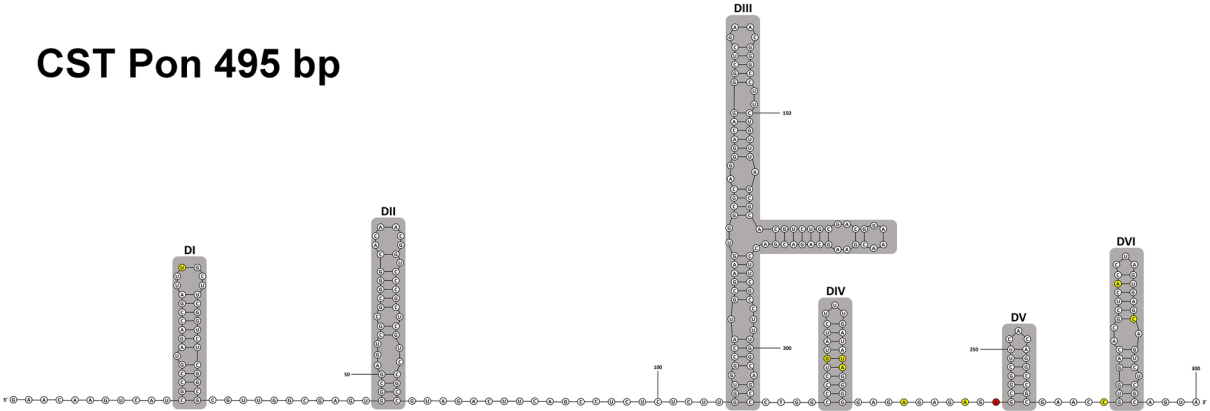

CST Pon 498 bp

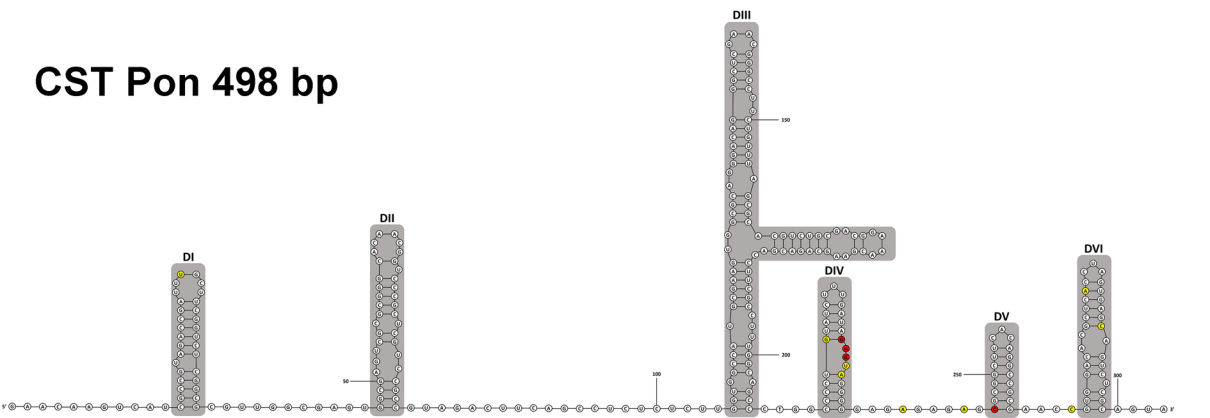

CGA Cad 496 bp

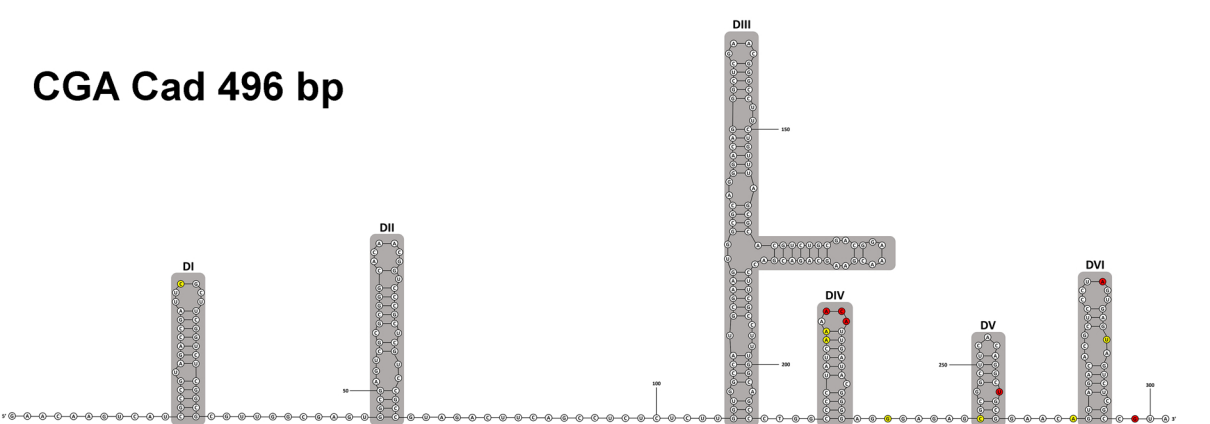

CGA Ita 498 bp

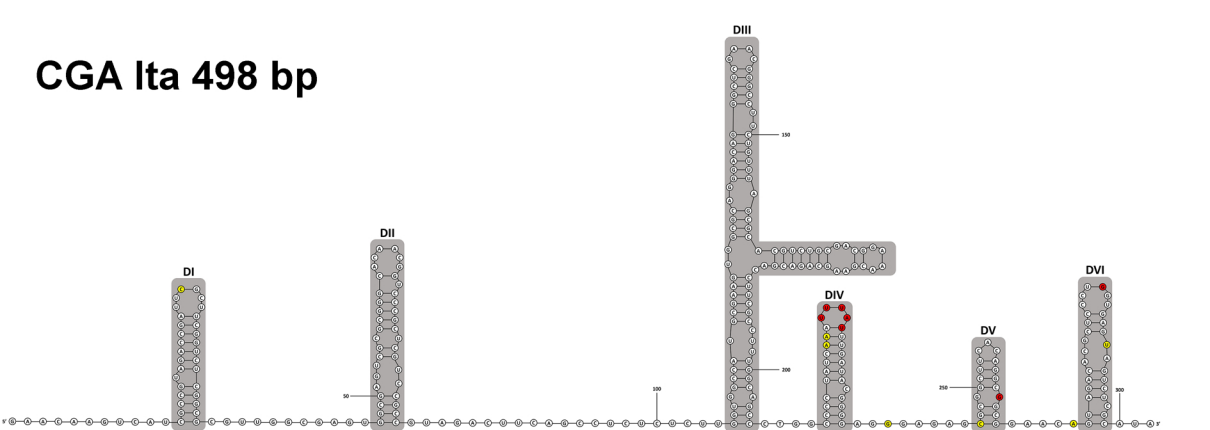

Supplement: Supplementary file 1 — PCR conditions used in the amplification of COI, 16S rRNA gene, ITS2, histone H3 and 5S and 28S rRNA genes. [file 7638790.f1.pdf]
